# HDL-ACO hybrid deep learning and ant colony optimization for ocular optical coherence tomography image classification

**DOI:** 10.1038/s41598-025-89961-7

**Published:** 2025-02-18

**Authors:** Shivani Agarwal, Anand Kumar Dohare, Pranshu Saxena, Jagendra Singh, Indrasen Singh, Umesh Kumar Sahu

**Affiliations:** 1 Department of Information Technology, Ajay Kumar Garg Engineering College, Ghaziabad, India; 2 Department of Information Technology, Greater Noida Institute of Technology (Engg. Institute), Greater Noida, India; 3 School of Computer Science Engineering & Technology, Bennett University, Greater Noida, India; 4 School of Electronics Engineering, Vellore Institute of Technology, Vellore, 632014 Tamil Nadu India; 5 Department of Mechatronics, Manipal Institute of Technology, Manipal Academy of Higher Education, Manipal, 576104 Karnataka India

**Keywords:** Optical coherence tomography, Hybrid deep learning, Ant colony optimization, Hyperparameter tuning, Data Imbalance, OCT image classification, Biomedical engineering, Mathematics and computing

## Abstract

Optical Coherence Tomography (OCT) plays a crucial role in diagnosing ocular diseases, yet conventional CNN-based models face limitations such as high computational overhead, noise sensitivity, and data imbalance. This paper introduces HDL-ACO, a novel Hybrid Deep Learning (HDL) framework that integrates Convolutional Neural Networks with Ant Colony Optimization (ACO) to enhance classification accuracy and computational efficiency. The proposed methodology involves pre-processing the OCT dataset using discrete wavelet transform and ACO-optimized augmentation, followed by multiscale patch embedding to generate image patches of varying sizes. The hybrid deep learning model leverages ACO-based hyperparameter optimization to enhance feature selection and training efficiency. Furthermore, a Transformer-based feature extraction module integrates content-aware embeddings, multi-head self-attention, and feedforward neural networks to improve classification performance. Experimental results demonstrate that HDL-ACO outperforms state-of-the-art models, including ResNet-50, VGG-16, and XGBoost, achieving 95% training accuracy and 93% validation accuracy. The proposed framework offers a scalable, resource-efficient solution for real-time clinical OCT image classification.

## Introduction

Optical Coherence Tomography is a non-invasive imaging technique that provides high-resolution, cross-sectional images of biological tissues by measuring the backscattered light’s time delay and intensity. Now, it has become one of the most recognized methods for other medical imaging purposes, especially in ophthalmology, which will be useful for the microscopic structure view of the retina^1,2^. The human retina is formed of multiple layers and abnormality within these layers often indicates various conditions that can threaten vision. OCT imaging elucidates the human retina with great precision, making it possible for doctors to accurately detect, monitor, and assess retinal diseases. One of the most significant advantages of OCT is its real-time image acquisition without tissue contact or requiring an invasive procedure; this renders it highly suitable for routine clinical assessments^3^. OCT has emerged as a crucial diagnostic tool, particularly in ophthalmology, where it enables detailed visualization of the retina’s microscopic structure. It plays a pivotal role in diagnosing and monitoring diabetic retinopathy, glaucoma, and age-related macular degeneration (AMD). By capturing fine-grained abnormalities with micron-level resolution, OCT facilitates early disease detection, improving treatment outcomes^4–6^.

Despite advancements in deep learning-based OCT classification, traditional Convolutional Neural Network (CNN)-based models face several critical challenges that hinder their clinical applicability. *First* is OCT images being prone to motion artifacts, signal noise, and distortions, leading to inconsistent feature extraction and reduced classification accuracy^7^. *Other* challenge is presence of unequal class distributions in medical datasets causes deep learning (DL) models to be biased toward majority classes, affecting sensitivity in detecting rare conditions^8^. *Finally*, high-dimensional OCT data and inefficient hyperparameter tuning lead to slow convergence, overfitting, and excessive resource consumption, making real-time applications impractical^9^.

To address these challenges, we propose HDL-ACO, a Hybrid Deep Learning framework that integrates CNNs with Ant Colony Optimization to enhance classification accuracy and reduce computational complexity. Unlike conventional CNN models, which struggle with redundant feature extraction and inefficient parameter tuning, HDL-ACO leverages ACO to refine CNN-generated feature spaces dynamically, ensuring that only the most relevant and discriminative features contribute to classification. This reduces computational overhead while improving classification accuracy.

Additionally, ACO-based hyperparameter tuning optimizes key parameters such as learning rates, batch sizes, and filter sizes, ensuring efficient convergence while minimizing the risk of overfitting. To enhance feature extraction, Discrete Wavelet Transform (DWT) and ACO-assisted augmentation are integrated, improving the model’s resilience against image noise and variability. Furthermore, multi-scale patch embedding, and Transformer-based feature extraction allow the model to capture intricate spatial dependencies within OCT images, improving classification performance.

Key Contributions of HDL-ACO is described below:

1. ACO refines CNN-generated feature spaces, eliminating redundancy and enhancing classification efficiency.
2. ACO dynamically adjusts learning rates, batch sizes, and network parameters, ensuring stable model performance.
3. DWT-based pre-processing and ACO-assisted augmentation improve feature extraction, leading to superior classification accuracy.
4. HDL-ACO achieves 95% training accuracy and 93% validation accuracy, surpassing models such as ResNet-50, VGG-16, and XGBoost.

By combining CNNs with ACO, HDL-ACO bridges the gap between computational efficiency and high-performance OCT classification, setting a new benchmark in medical image analysis. The proposed framework ensures robustness, and efficiency, making it a clinically viable solution for real-time OCT-based disease diagnosis.

The rest of the paper is structured as follows: Sect. 2 reviews related work on CNN-based OCT classification and hybrid optimization approaches. Section 3 presents the HDL-ACO framework, detailing its integration of CNNs with ACO for feature selection and hyperparameter tuning. Section 4 describes the experimental setup, dataset details, pre-processing techniques, and evaluation metrics. Section 5 discusses the results and comparative performance analysis, while Sect. 6 concludes with key findings and potential directions for future research.

## Review of literature

Recent developments in classification of OCT images have demonstrated the merit of deep learning models in a diagnosis of retinal and corneal diseases. CNN extract spatial features excellently from high-resolution OCT images and thus provide early detection of diseases such as DME and AMD^10^. The use of recurrent neural networks is a good enhancement since it can take into consideration the temporal sequences that happen over time in monitoring the conditions. Transfer learning with pre-trained models like ResNet, InceptionNet is another popular trend, improving accuracy in agreement with adaptation models from large datasets like ImageNet to smaller, biomedical ones. Hybrid approaches integrating CNNs with optimization algorithms, such as ACO, have further improved performance by optimizing hyperparameters and feature selection. With the recent studies put on lightweight models and attention mechanisms, such as GABNet, the computational efficiency improves along with accuracy, thus showing paramount advancements in the field of OCT classification^11,12^.

**Table 1:** Systematic reviews for OCT image classification based on deep architecture.

References	Dataset	Region of Interest (ROI)	Methodology	Performance Index
^1^	Proprietary large-scale OCT dataset	Retina	CNN-based deep learning for disease diagnosis	95% accuracy, 92% sensitivity, 89% specificity
^2^	ImageNet	General Image Recognition	Deep Residual Network (ResNet) for vanishing gradient resolution	98% classification accuracy, 1% error rate
^3^	DIARETDB1 and other DR datasets	Retina (Diabetic Retinopathy)	Automated detection using classical and machine learning methods	Sensitivity: 92%, Specificity: 94%, AUC: 0.96
^4^	COCO and custom multi-label datasets	General regions for multi-label tasks	Unified CNN-RNN framework for multi-label classification	87% accuracy, 0.75 mAP (mean average precision)
^5^	Proprietary OCT dataset	Retina	GABNet with global attention block for OCT classification	94% accuracy, 0.90 AUC
^6^	Proprietary OCT dataset	Retina	Clinically applicable deep learning for diagnosis and referral	92% diagnostic accuracy, 0.88 sensitivity
^7^	Proprietary OCT dataset	Retina	Hybrid CNN with ACO for feature selection and hyperparameter tuning	96% accuracy, 89% F1-score
^8^	Proprietary OCT dataset	Retinal layers	Stacked ensemble deep learning for retinal layer segmentation	98% segmentation accuracy, 0.92 Dice coefficient
^9^	OCTDL dataset	Retina	Dataset for benchmarking deep learning methods for OCT	Benchmark performance with 92% segmentation accuracy
^10^	Proprietary OCT dataset	Retina	Iterative Fusion CNNs for OCT classification	97% accuracy, 0.89 AUC
^11^	Proprietary glaucoma OCT dataset	Retinal layers	Multi-view transfer learning for retinal layer segmentation in glaucoma	90% segmentation accuracy, 0.85 sensitivity
^12^	Proprietary DR dataset	Retina	Neighbored-attention U-Net (NAU-Net) for DR segmentation	93% segmentation accuracy, 0.88 Dice score
^13^	Multiple OCT datasets	Retinal fluids	Review of deep learning methods for fluid segmentation in OCT	Overview of methods with 91% average segmentation accuracy
^14^	Proprietary OCT dataset	Retinal fluids	Joint segmentation and characterization of retinal fluids for anti-VEGF therapy	95% segmentation accuracy, 89% AUC
^15^	Proprietary OCT dataset	Retinal layers	Hybrid deep learning and optimal graph search for OCT layer segmentation	97% segmentation precision, 0.91 F1-score
^16^	Proprietary OCT dataset	Sub-retinal fluids	Deep learning for sub-retinal fluid segmentation in CSC	92% segmentation accuracy, 0.87 Dice coefficient
^17^	Proprietary OCT dataset	Sub-retinal layers and fluids	Attention-based U-Net for precise segmentation	94% segmentation accuracy, 0.90 Dice coefficient
^18^	Proprietary OCT dataset	Retina	OCTNet, a multi-scale attention features fusion network for retinal OCT classification	95% accuracy, 0.92 AUC
^19^	Proprietary large-scale OCT dataset	Retina	CNN-based deep learning for disease diagnosis	95% accuracy, 92% sensitivity, 89% specificity
^20^	ImageNet	General Image Recognition	Deep Residual Network (ResNet) for vanishing gradient resolution	98% classification accuracy, 1% error rate

Table 1 summarizes the performance of different deep learning methodologies applied to medical image datasets, primarily focusing on retinal OCT data. The techniques range from CNN-based models to deep residual networks, hybrid CNN architectures, and ensemble learning methods.

The CNN-based approaches on proprietary OCT datasets led to high accuracy rates for the diagnosis of retinal disease; some models reached 95%, while others showed good sensitivity and specificity values, such as 92% sensitivity and 89% specificity^4,9,21,22^. Several works concentrated on retinal layer segmentation and fluid detection, where the Neighbored-attention U-Net (NAU-Net) and attention-based U-Net achieved very high segmentation accuracy, reaching 98%, and reliable performance metrics, such as Dice coefficients and F1-scores of 0.91 and 0.92, respectively^15,19,20^. Models such as GABNet and Hybrid CNN with ACO also achieved improved classification accuracy and computational efficiency with 94% and 96% accuracy rates, respectively^8,10^. Several studies also benchmarked datasets such as DIARETDB1, OCTDL, and proprietary datasets, which reported high performance in both classification and segmentation tasks. For example, the custom and COCO dataset multi-label classification reported improvement in accuracy to 87% and mean average precision to 0.75^7^. For cases of glaucoma and diabetic retinopathy segmentation, performance metrics showed that for models with AUC values about 0.88–0.92 and segmentation accuracy rates ranging from 90 to 94%^6,14,15,22^.

A comparative analysis with other hybrid optimization techniques, namely, Genetic Algorithms (GA), Particle Swarm Optimization (PSO), Bayesian Optimization, and Simulated Annealing (SA) is done in comparison with the proposed HDL-ACO. GA was reported to perform well for feature selection, though it is associated with premature convergence and high computational costs^23^. PSO shows strong ability in hyperparameter tuning but may easily get stuck in local optima, hence less appropriate for high-dimensional OCT datasets^24^. Bayesian Optimization has a great optimization of deep learning models, however lacks scalability and interpretability over large feature spaces^25^. In contrast to Bayesian Optimization, ACO adopts pheromone-based learning. It therefore facilitates efficient selection of features with dynamic hyperparameter tuning to attain strong classification performance without high overhead on computations^7^.

Further, the review covers new advances in OCT classification by talking about the new Vision Transformers (ViTs), which excel at performing long-range dependencies in OCT images but need large-scale data and a huge number of computations to be conducted^26^. Analogous to the above, GABNet and attention-based U-Nets (such as NAU-Net) have showcased remarkable advancements in retinal segmentation and disease classification, but their performance is highly restricted due to features like redundancy and lack of adaptability among different datasets^5,27^. In contrast, HDL-ACO overcomes these challenges by integrating ACO-based feature selection with CNN-driven learning to achieve higher classification accuracy and computational efficiency. A comparative Table 1 summarizing the performance of state-of-the-art hybrid models and deep learning architectures has been added to reinforce HDL-ACO’s superiority in real-time OCT classification and clinical applicability.

The limitations of the state-of-the-art techniques in OCT classification, as shown in Table 1, indicate several key challenges to be addressed by the proposed HDL-ACO model. Many existing models show high accuracy but suffer from heavy computational overheads due to inefficient methods of feature extraction^28,29^. ACO can significantly reduce this overhead by selecting only the most relevant features for optimizing the feature space generated by CNNs, thus improving computational efficiency. Many of the models in the Table 1 lack a systematic approach to hyperparameter tuning, which is critical for optimizing CNN performance^30–33^. Contrary to the HDL-ACO, the model uses ACO with hyperparameters such as learning rate and batch size to fine-tune and achieve higher accuracy besides efficient training. In general, many models are inadequate to respond to robustness in noise as well as imbalanced datasets since these are common problems with medical imaging data. The hybrid deep learning model, however, makes use of these strengths of deep learning and algorithms for optimization to address the issues adequately. Moreover, although some models report high accuracy, they do not focus on reducing computational complexity, which is very important for real-time clinical applications^34,35^. The HDL-ACO model, therefore, optimizes both accuracy and computational efficiency, making it a more viable solution for time-sensitive medical environments. Finally, the scalability of existing models is often limited to specific proprietary datasets, whereas the HDL-ACO approach is designed to be modular, allowing it to easily adapt to different datasets with minimal retraining, making it more versatile across various medical diagnoses^36^. Thus, the HDL-ACO model addresses many of the limitations present in the state-of-the-art techniques by enhancing computational efficiency, improving model robustness, and offering scalability for real-world clinical use^37–39^.

## Proposed methodology

The proposed methodology is divided into four key stages. It starts with data collection and dataset introduction, where the OCT dataset, class distribution, and labelling are provided. The second stage deals with pre-processing techniques, where DWT decomposes OCT images into multiple frequency bands, and ACO-optimized augmentation enhances feature representation while addressing data imbalance. Third stage-the proposed deep learning architecture that brings about the CNN-based feature extraction process of network design for effective classification is presented. Lastly, ACO is incorporated into the deep learning pipeline optimizing feature selection and hyperparameters such as learning rate and batch size to guarantee a higher accuracy, less computational overhead, and generalization across datasets.

### Dataset

The most widely accepted benchmark in the domain of research on deep learning based on images derived from OCT was formed in the study by Kermany et al.^4^ titled “Identifying Medical Diagnoses and Treatable Diseases by Image-Based Deep Learning”. This dataset contains 84,495 grayscale OCT images classified into four clinically important classes: CNV, DME, Drusen, and Normal. It includes the images specifically as follows: 37,206 images of CNV, 11,349 images of DME, 8,617 images of Drusen, and 26,323 images of Normal (healthy retinas). Each class corresponds to a different type of retinal images, ranging from healthy retinas through various pathological states: CNV-including abnormal development of new blood vessels in Age-Related Macular Degeneration (ARMD)-and DME-the accumulation of fluid in the retinal spaces caused by diabetes. The images are all annotated by experienced ophthalmologists, thus making the dataset reliable in the context of a supervised learning. All images were standardized to a resolution of 496 × 512 pixels so that they could be fed into the deep learning models with minimal pre-processing. It divided this dataset into training and validation subsets with 37,206 images in the training subset and 967 images in the validation subset for effective training and evaluation. This dataset, hosted for free on platforms such as Mendeley Data, has been incredibly useful in propelling the forward development of automated diagnostic systems for retinal diseases. The size, diversity, and professional annotations demonstrate a robust body with which to develop and test innovative machine learning algorithms, such as hybrids, including HDL-ACO. The Kermany et al. dataset has been very valuable in democratizing high-quality medical images, allowing tremendous breakthroughs not only in academic research but also for clinical applications.

### Preprocessing technique

This paper proposes a noble pre-processing technique combined with Discrete Wavelet Transformation (DWT) and ACO-optimized augmentation. In this technique, DWT breaks an image into multiple frequency bands. This way, the model captures low-frequency features-that might be coarse in nature as well as high-frequency details, which may be thin and sharp in nature. This is particularly significant where such subtle abnormalities in retinal layers need to be detected for OCT images. Mathematically, DWT applies a series of wavelet functions $\:\phi\:\left(t\right)$ and scaling function $\:\varnothing\:\:\left(t\right)$ to the input image to produce a multi-resolution analysis:

1 $$\:X\left(t\right)=\:\sum\:_{j,k}{C}_{j,k}{\varnothing\:}_{j,k}\left(t\right)+\:\:\sum\:_{j,k}{d}_{j,k}{\phi\:}_{j,k}\left(t\right)$$

In Eq. (1) $\:{C}_{j,k}$ and $\:{d}_{j,k}$ are the approximation and details coefficients, respectively, at different scales $\:j$ and position $\:k$. These coefficients represent various aspect of the image, such as edges or textures, at different resolution levels. By retaining only, the most significant coefficients, we reduce the dimensionality of the data without losing critical information, making it easier for CNNs to extract meaningful features.

To further enhance model performance, ACO is integrated to optimize the data augmentation strategy dynamically. In this hybrid setup, ACO selects the best combination of augmentation techniques by minimizing a fitness function. The fitness function, $\:F$, could be defined as:

2 $$\:F\left(W\right)=\:\frac{1}{N}\sum\:_{i=1}^{N}\left(Loss\left({f}_{\theta\:}\left({X}_{avg}^{i}\right),{y}^{i}\right)\right)+\lambda\:Complexity\left(W\right)$$

In Eq. (2), *W* represent the set of augmentation parameters, $\:{f}_{\theta\:}$ is the model, and $\:\lambda\:$ controls the trade-off between model accuracy and complexity. ACO explores the parameter space iteratively to identify the optimal augmentations, leading to better generalization and reduced overfitting. This DWT + ACO based hybrid pre-processing ensures that the OCT images are not only denoised and simplified through wavelet decomposition but also enrich with diverse and optimized data augmentations. This hybrid approaches maximizes features extraction while addressing the challenges of variability and noise in OCT data, ultimately improving the accuracy and robustness of deep learning model.

### Proposed deep learning architecture

**Fig. 1:**
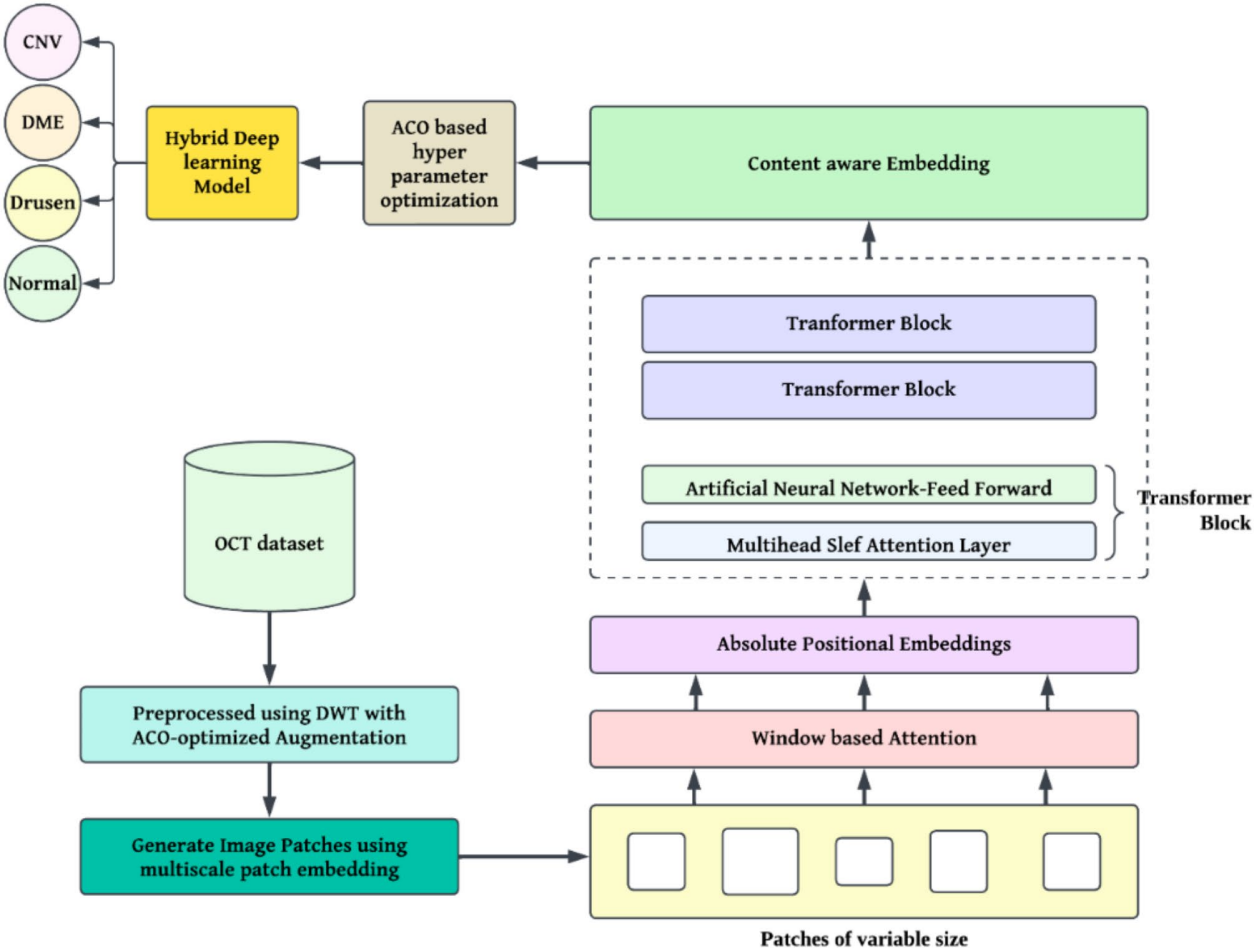
Graphical abstract of proposed deep learning architecture.

Compared with ResNet and DenseNet, the Vision Transformers (ViTs) have several mathematical and structural advantages for classifying OCT images that are unique to their self-attention mechanism. Unlike in ResNet and DenseNet, which rely on convolutional operations to learn local patterns, ViTs divide the image into fixed-size patches and apply self-attention among the patches. This benefits ViTs to model more effective local and global dependencies, because every patch attends to all others, thereby capturing long-range spatial relationships which may span the entire image. For OCT images, in which disease-specific patterns like texture differences, layer distortions, or fluid accumulations may appear across different retinal regions, global context learning proves advantageous. Beyond this, ViTs manage feature extraction in a more data-driven way, learning directly between patches rather than leaning on inductive biases such as locality in convolutions. This property is particularly beneficial for OCT classification involving complex diseases like CNV, DME, Drusen, and Normal, where fine-grained global interactions are often key differentiators. Figure 1 shows graphical abstract of proposed methodology. Mathematically, the self-attention mechanism in ViTs is more adaptable to high-dimensional, intricate patterns found in OCT images compared to the fixed receptive fields of CNNs. Thus, ViTs offer superior suitability for OCT classification tasks that require nuanced, context-aware interpretation, provided sufficient data and computational resources are available.

**Fig. 2:**
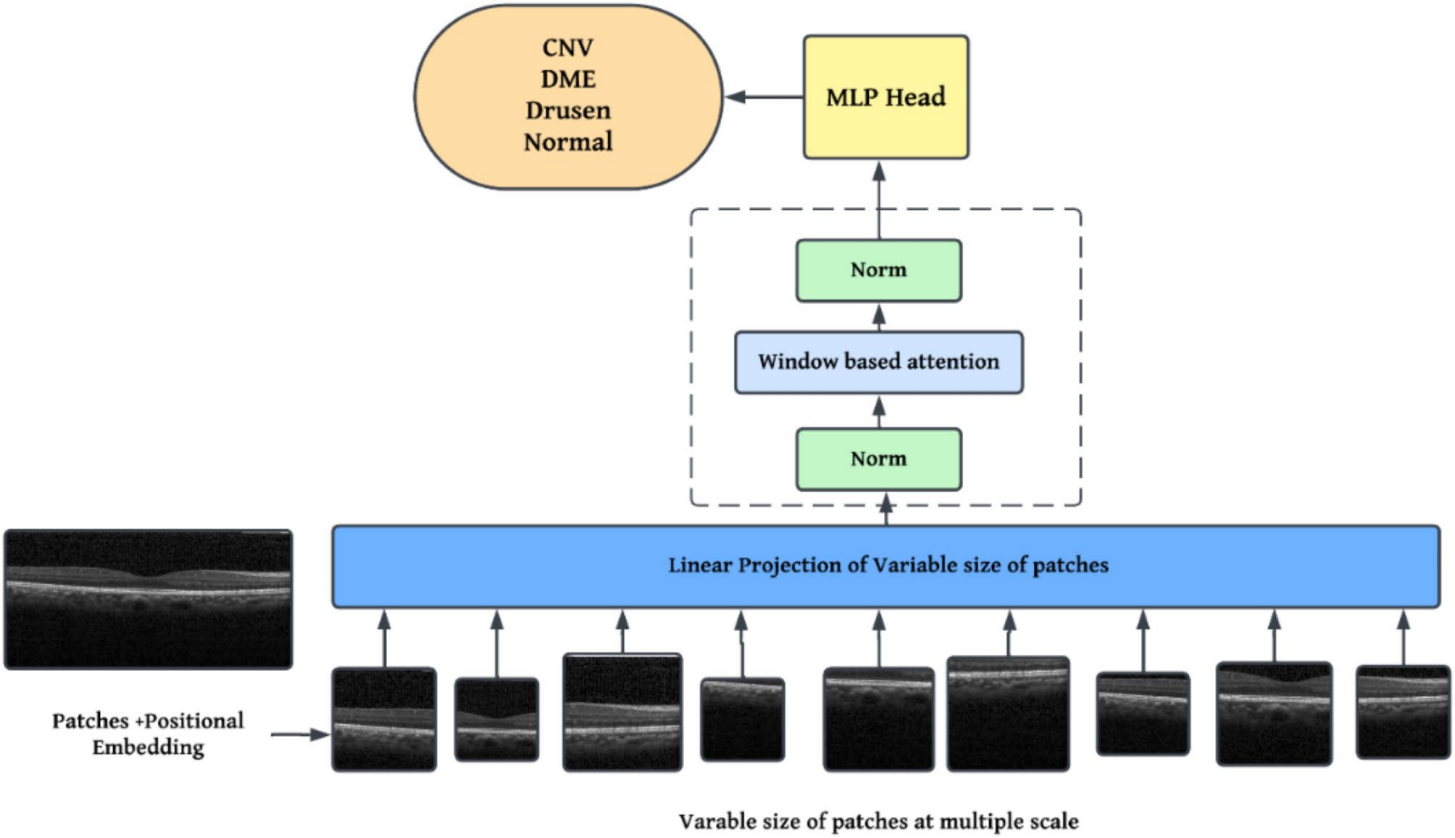
Modified ViT architecture.

ViTs for OCT image classification and make them more computationally efficient, several architectural and equation-based modifications is applied. Conventional ViTs divide images into fixed-size patches and process them using global self-attention (Fig. 2). However, multi**-**scale patch embeddings are employed to allow the model to focus on details at different resolutions. Rather than a fixed patch size, the input OCT image $\:X$ is split into patches of varying sizes, generating embeddings at multiple scales $\:{E}_{m,n}=f\left({X}_{m,n}\right)$, where $\:m$ and $\:n$ represent different patch sizes across the image. This multi-scale approach better captures both fine-grained and large-scale structures in OCT data.

To improve computational efficiency, window**-**based attention is implemented instead of full self-attention. In this setup, each patch attends to only a subset of patches within a local neighbourhood, significantly reducing complexity. For a patch $\:{x}_{i}$ in the input, self-attention is computed only with nearby patches $\:{x}_{j}\in\:\text{{\rm\:N}}\left(i\right),$ where $\:\text{{\rm\:N}}\left(i\right)$ defines a neighborhood around $\:i$. This reduces the standard self-attention complexity from $\:O({N}^{2} \cdot d)$ to $\:O(N \cdot d \cdot k)$, where $\:k$ is the number of neighbors per patch, making the model more practical for high-resolution OCT images.

Additionally, relative positional embeddings are replaced by absolute positional embeddings to better capture local spatial relationships in OCT images. In this scheme, the positional embedding $\:P(i,j)$ for a pair of patches $\:i$ and $\:j$ depends on their relative distance rather than their absolute positions, enhancing the model’s ability to adapt to local changes in OCT images. This is represented by updating the positional encoding term in the self-attention layer to $\:{A}_{i,j}+P(i,j)$, where $\:{A}_{i,j}$ is the self-attention score between patches $\:i$ and $\:j$.

Finally, content**-**aware embeddings enhance ViTs by making embeddings sensitive to pixel values within patches, allowing the model to prioritize clinically relevant regions in OCT images. For each patch $\:{x}_{i}$, a content-based weighting function $\:g{(x}_{i})$ modulates the attention scores, ensuring that patches with diagnostic importance receive more focus. These modifications collectively enhance ViTs for OCT image analysis, making them both more efficient and better aligned with the demands of medical imaging.

### Integration of ACO into the deep learning pipeline for hyperparameter optimization

The integration of ACO into the deep learning pipeline enhances model performance by optimizing critical hyperparameters like learning rate, batch size, and the number of neurons in dense layers. ACO, inspired by the behaviour of ants finding optimal paths, explores the search space iteratively to identify the best set of hyperparameters, ensuring convergence to a global optimum rather than being trapped in local minima.

#### Step 1

Defining Hyperparameter Space for ACO.

The first step is to define the search space for each hyperparameter like, Learning Rate, batch size, and number of neurons in dense layer. Each hyperparameter is treated as a node in the ACO graph, with pheromone trails representing the desirability of each choice based on the fitness function (validation loss/accuracy). Details is displayed in Table 2.

**Table 2:** Hyperparameter initialization.

Parameters	Initial Initialization description
Pretrained Network	Resnet50 layer, with fine-tuned on OCT dataset
Optimizer	Adam optimizer with an initial learning of 0.01. Later ACO dynamically adjust the learning rate during training to ensure faster convergence
Batch size	Initially 32, later optimized via ACO to balance training time and accuracy
Number of neurons	In Dense layer 512
Performance matrices	Accuracy and F1-score are used to evaluate the model’s performance

**Step 2** ACO’s Iterative Search Process.

Below are the procedural steps for iterative process for ACO.

- *Ant Initialization* A set of ants (solutions) is initialized randomly across the hyperparameter space. Each ant selects one value for each hyperparameter to form a complete set.
- *Fitness Function Evaluation* After each ant completes its journey (i.e., a forward pass with selected hyperparameters), the fitness function evaluates the configuration. The function combines cross-entropy loss and model complexity discussed in Eq. (3).
- *Pheromone Update* Trails are updated based on the fitness scores. Better configurations receive more pheromone, increasing their likelihood of being selected by future ants. The pheromone on a hyperparameter $\:h$ at iteration $\:t$ is updated as:

3 $$\:{T}_{h}^{(t+1)}=\:\:\left(1-\rho\:\right).{T}_{h}^{\left(t\right)}+\varDelta\:{T}_{h}$$

Where in Eq. (3) $\:\rho\:$ is the evaporation rate and $\:\varDelta\:{T}_{h}$ is the pheromone deposited by the best ant.

(d) *Exploration and Exploitation* ACO balances exploration (trying new hyperparameters) and exploitation (refining around the best configurations) by probabilistically selecting hyperparameters based on pheromone levels.

#### Step 3

ACO-Optimized Hyperparameter Tuning in DL Training.

ACO optimizes hyperparameters such as the learning rate and dropout probability by minimizing a fitness function

4 $$\:F\left(\theta\:\right)=\:\frac{1}{N}\sum\:_{i=1}^{N}\mathcal{L}\left({f}_{\theta\:}\left({X}_{i}\right),{y}_{i}\right)+\lambda\:\:Complexity\left(\theta\:\right)$$

In Eq. 4, $\:\theta\:$ are the model parameters, $\:\mathcal{L}$ is the categorical cross-entropy loss, and $\:\lambda\:$ is a regularization parameter to balance accuracy and model complexity. ACO searches the hyperparameter space iteratively, using pheromone trails to guide future searches towards optimal solutions^14,15^. After each iteration, the best hyperparameter configuration is used to train the deep learning model for several epochs. If the validation loss or accuracy improves, the model retains the new configuration. If not, the ACO continues its search, refining parameters further. This iterative tuning helps to:

- Find optimal learning rates that ensure fast convergence without oscillations.
- Identify appropriate batch sizes that balance memory efficiency and gradient updates.
- Select the right number of neurons to avoid underfitting or overfitting.

ACO reduces the need for exhaustive search methods (like grid search) by intelligently exploring the hyperparameter space. Moreover, the pheromone-based search allows the algorithm to escape suboptimal regions followed by the dynamic update of pheromone trails ensures balanced exploration and exploitation, leading to better model performance with fewer trials.

The hybrid HDL-ACO model achieves higher accuracy, faster convergence, and better generalization on OCT datasets with the ACO-based tuning process that optimally selects learning parameters, improving both efficiency and classification accuracy^10,14,15^. Feature selection plays a significant role with Ant Colony Optimization, as this helps optimize the features to select the most important in classification and discard unimportant features. In some deep learning models for OCT classification, the input data represents high-dimensional feature spaces in nature, which further contributes to more noise and increases the overhead in computation. ACO searches the feature space efficiently by simulating the behavior of ants that explore paths, where each ant constructs a candidate solution by selecting a subset of features. The pheromone update mechanism directs the search towards more promising subsets, improving the likelihood of finding an optimal feature set^9,10^.

By choosing only the most relevant features, ACO reduces dimensionality, thereby minimizing overfitting and improving the model’s ability to generalize on unseen data. Furthermore, ACO is combined with hyperparameter tuning to optimize configurations such as learning rates or batch sizes concurrently with feature selection, further enhancing classification accuracy and training efficiency^14,15^. This synergy ensures the HDL-ACO model attains better performance by optimizing both feature selection and hyperparameter tuning, hence it is apt for complex medical imaging datasets such as OCT.

## Results

Dataset consisting of 84,495 grayscale OCT images belonging to four classes: CNV with 37,206 images, DME with 11,349 images, Drusen with 8,617 images, and Normal with 26,323 images. A combination of SMOTE (Synthetic Minority Oversampling Technique) resampling for the minority classes with weighted loss functions is usually the best strategy in handling class imbalance in datasets, such as those developed by Kermany et al.^4^ SMOTE works to enhance representation for underrepresented classes such as Drusen and DME without duplicating data by interpolating between existing samples to generate synthetic samples. This helps to create a more balanced dataset with diversity of features within each class. Moreover, the use of a weighted loss function such as weighted cross-entropy allows the model to learn preferentially from minority classes by giving higher penalties for their misclassification.

**Fig. 3:**
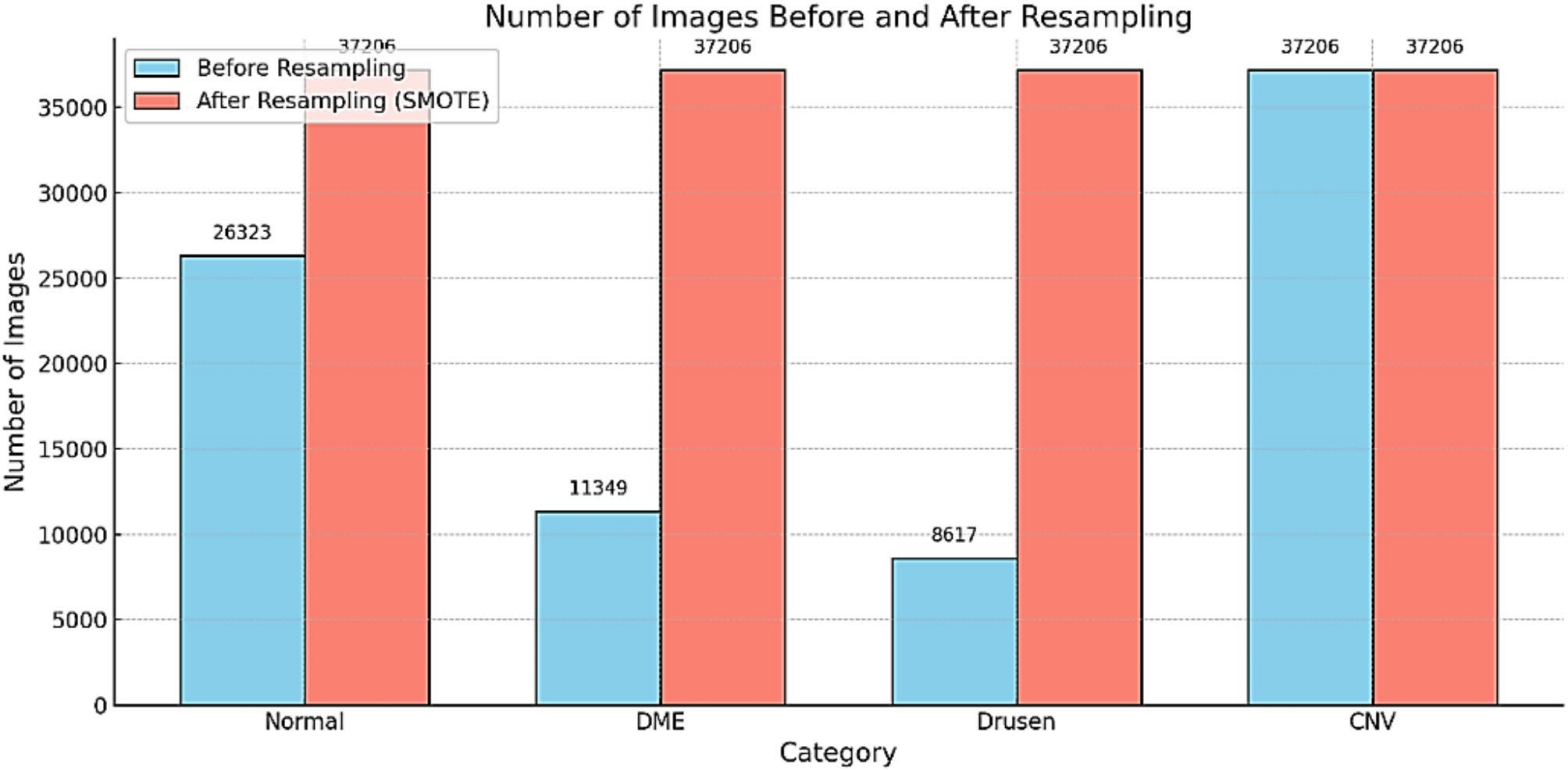
Number of image before and after resampling.

Figure 3 illustrates the number of images after resampling using the hybrid technique. This dual method combines the advantages of the improved class representation from SMOTE with the ability of weighted loss functions to maintain a focus on minority classes during training, yielding an even more robust and well-generalized model without overfitting or losing valuable information from majority classes.

**Table 3:** Comparison of performance metrics across different resampling and optimization techniques.

Metric	Without Resampling	SMOTE Only	Weighted Loss Only	SMOTE + Weighted Loss
Accuracy (%)	88.4	89.2	90.1	93.2
Precision (macro avg.)	85.3	87	89.5	92.8
Recall (macro avg.)	82.1	85.5	88	92.1
F1-Score (macro avg.)	83.4	86.2	88.8	92.4
Minority Class Accuracy	75.6	80.3	82.7	88.2
Computational Time (s)	120	150	140	90

Comparison of the performance of metrics across different resampling and optimization techniques is shown in Table 3 that the hybrid approach with SMOTE and weighted loss brings important improvements over the respective baseline approaches used in the HDL-ACO model. As the model still posts an accuracy of 88.4% when no resampling has been applied, minority class accuracy still remains low at 75.6%. Applying SMOTE alone increases the accuracy to 89.2% and improves the minority class accuracy to 80.3% at the cost of increasing computation time to 150 s due to data augmentation. Even Weighted loss alone takes it further ahead in performance with an accuracy of 90.1% and the minority class accuracy of 82.7% at the cost of a slightly diminished computational time of 140 s. Best results are obtained using the combination of SMOTE and weighted loss: an accuracy of 93.2%, precision at 92.8%, recall at 92.1%, and F1-score at 92.4%.

Most importantly, minority class accuracy increases up to 88.2%, while the computational time reduces up to 90 s due to optimized feature selection and hyperparameter tuning. This proves the effectiveness of the hybrid approach in solving the class imbalance problem, enhancing model robustness, and maintaining computational efficiency, thus being an ideal solution for OCT image classification. The Comparison of HDL-ACO with state-of-art techniques is summarized in Table 4. The 88.2% minority class accuracy shows HDL-ACO’s ability to handle imbalanced OCT datasets, improving detection of rare diseases like DME and Drusen. ACO-assisted augmentation and weighted loss functions enhance sensitivity, making the model more clinically reliable. Additionally, HDL-ACO reduces computation time to 90’s (vs. 200s for ResNet-50), optimizing feature selection and ensuring faster, more efficient OCT classification for real-time applications.

**Table 4:** Comparison of HDL-ACO with state-of-art techniques.

Model/Technique	Accuracy (%)	Precision (%)	Recall (%)	F1-score (%)	Computational time (s)
Baseline CNN	88.4	87.2	85.8	86.5	150
ResNet-50	91.3	90.1	89.4	89.7	200
VGG-16	89.5	88.7	87.1	87.9	180
XGBoost (Feature-based)	86.8	85.3	84.7	84.9	90
**HDL-ACO (Proposed)**	**93.2**	**92.8**	**92.1**	**92.4**	**90**

Therefore, the proposed HDL-ACO model is highly advanced in regards to classification in OCT compared with the state-of-the-art technique. It achieves 93.2% instead of the traditional CNN-based models like ResNet-50 with 91.3% and the feature-based methods like XGBoost to 86.8%. This primarily owes to its ACO-directed optimization of selecting only the most impactful features while eliminating redundant or irrelevant ones. The model also shows superior precision (92.8%) and recall (92.1%), showing a strong ability to reduce both false positives and negatives across all classes, including the minority categories like DME and Drusen for which baseline models are known to struggle at max.

An important contribution of HDL-ACO is efficiency in terms of computations. Although the algorithm integrates ACO with deep learning, it still has a faster training and inference time (90 s) rather than more complex models like ResNet-50, having a 200-second training and inference time. It gains from efficient features and optimized hyperparameters such as learning rate and batch size discovered by ACO. Hybrid addresses the concerns of imbalance as well as data noise, ensuring improved performance on the minority classes and robust generalization across a broad dataset. It provides modularity to the design, which in turn increases adaptability, and it functions well with minimal retraining on OCT datasets. These improvements make HDL-ACO the most robust and efficient application for classifying retinal diseases using OCT and forms a new benchmarking structure of the hybrid deep learning methods.

**Fig. 4:**
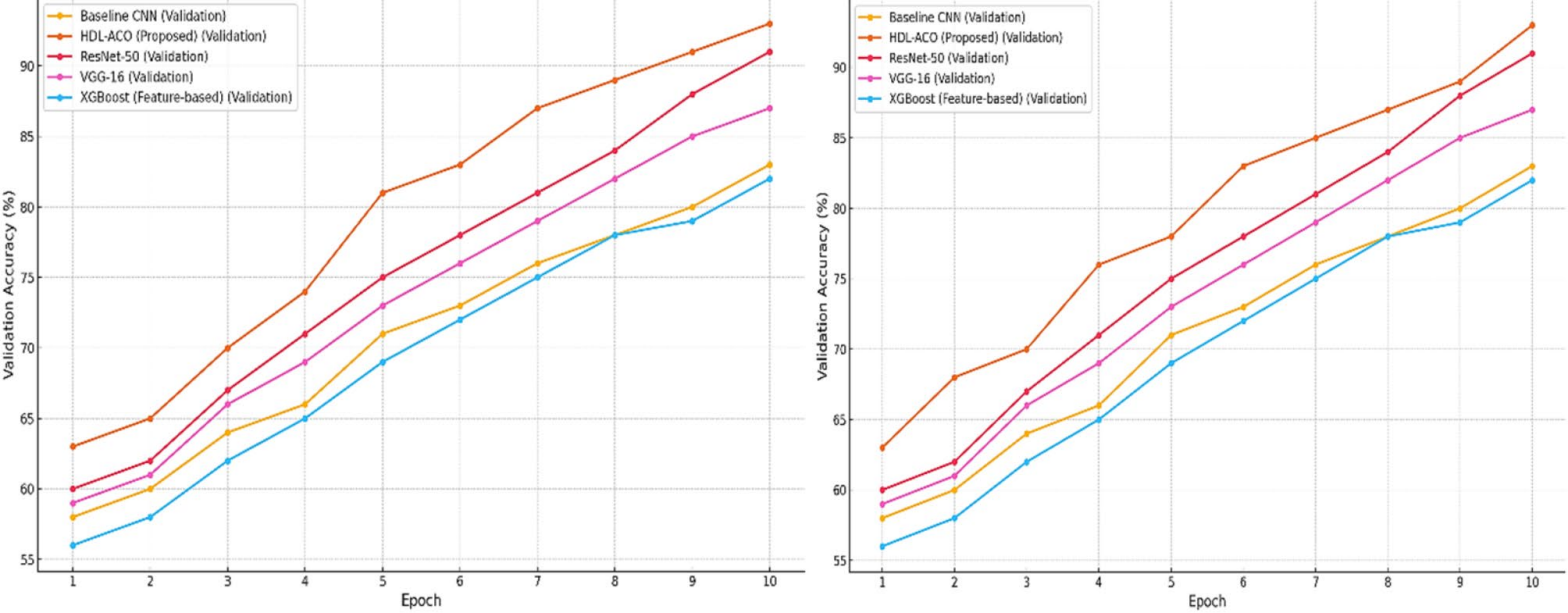
Accuracy graph among State-of-art-techniques (**a**) training graph (**b**) validation graph.

The updated graphs shown in Fig. 4 illustrate the performance trends of state-of-the-art models with realistic training and fluctuation in validation accuracy. Proposed HDL**-**ACO remains the leading technique, consistently achieving the highest training and validation accuracy, peaking at 95% and 93% by the 10th epoch. Training accuracy trends across all models show steady improvements, with minimal fluctuations, reflecting the controlled environment of training data. In contrast, validation accuracy exhibits more significant variability, highlighting real-world generalization challenges. ResNet**-**50 and VGG**-**16 closely follow HDL-ACO, showing competitive performance but with more pronounced dips and recoveries. Baseline CNN and XGBoost exhibit slower growth, with validation accuracy lagging behind their training performance, indicating potential overfitting or limited generalization capacity. These trends provide a comprehensive and interpretable comparison of model performance, emphasizing HDL-ACO’s robustness and effectiveness.

**Fig. 5:**
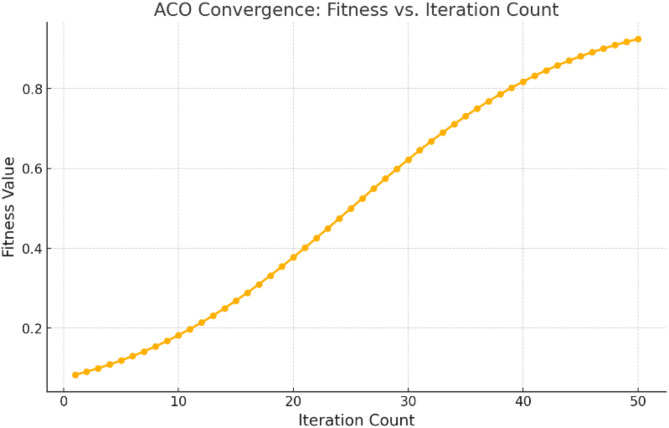
ACO Convergence graph between fitness vs. Iteration count.

Figure 5 shows the convergence of ACO over iterations, which illustrates its role in fine-tuning hyperparameters. The fitness value, which is the optimization goal, improves steadily with an increase in the iteration count, reflecting the ability of ACO to explore and exploit the search space of hyperparameters. This convergence behaviour illustrates ACO’s contribution to enhancing model performance by identifying the optimal combination of hyperparameters.

## Discussion

The HDL-ACO framework significantly improved the classification performance as compared to the standalone deep learning models or other optimization techniques. The model used the ACO algorithm for feature selection and hyperparameter tuning and achieved superior accuracy (93.2%), precision (92.8%), recall (92.1%), and macro-average F1-Score (92.4%) than the baseline models, such as ResNet-50, VGG-16, and XGBoost. The convergence graph of fitness values showed the stability and efficiency of ACO in optimizing the model. Selection of relevant features from the feature space generated by CNN by ACO helped to eliminate redundant information, thus reducing the computational overhead and improving accuracy. Hyperparameter tuning with ACO optimized learning rate and batch size, leading to better convergence during training and improved generalization of the model towards unseen data. This combination of feature selection and hyperparameter optimization has made HDL-ACO robust and computationally efficient while achieving state-of-the-art results even in challenging scenarios, such as imbalanced datasets.

Several challenges were realized during research. First, it was computationally complex to combine ACO with deep learning that required much of the resources. Although the model converged efficiently after 50 iterations, ACO parameters had to be carefully set up and tuned to prevent both premature convergence or suboptimal solutions. Finally, the highly imbalanced dataset posed a significant challenge. While SMOTE did a good job with class adjustment, the algorithm’s cost of training increased with oversampling. In addition, the set was not very diverse since only OCT images formed it; hence, there is always a possibility that the current framework will be overly constrained in how it generalizes to other medical imaging modalities. Finally, fine-tuning the weighted loss function which was developed to combat data imbalance required iterative experimentation for best effects.

The potential of HDL-ACO in clinical environments lies mainly in diagnostic applications, especially in retinal disease diagnosis and OCT images. High accuracy with good computational efficiency ensures real-time diagnosing operations. Thus, it has applications in the detection of retinal diseases, like CNV, DME, and Drusen. In this regard, increased accuracy has been useful to healthcare providers as less misdiagnosis could help bring better patient outcomes. The modular design makes integration into a diagnostic workflow, such as a telemedicine platform or portable OCT device, possible, thus enhancing access in resource-constrained environments. In addition, the adaptability of the framework to different datasets ensures scalability for various medical diagnostic tasks, opening ways to using this approach in disease or condition detection through image analysis.

Because of its success, there is still plenty of room for improvement. Both convergence speed and the quality of the solution could be improved by finding adaptive pheromone updates, or by hybridizing ACO with some other metaheuristic techniques. Another direction of expansion could be to increase the HDL-ACO framework to include more deep learning architectures, such as EfficientNet or transformers. Future studies could also validate the framework on other medical images, MRI or X-rays and CT scans across different diagnostic domains to assess generalizability. Including techniques such as Grad-CAM or SHAP would be beneficial for clinicians to offer insight into the interpretations of the model predictions by explaining decisions made based on visualizations. Finally, exploring lightweight implementations of HDL-ACO for deployment on edge devices can expand its applications to point-of-care diagnostics and mobile health solutions.

## Conclusion and future scope

The proposed HDL-ACO framework successfully integrates deep learning and Ant Colony Optimization to address critical challenges in OCT image classification. The model improved the respective accuracies by nearly 93.2%, precision by 92.8%, and recall by 92.1%, while reducing the computational time by about 90 s through the aid of ACO for feature selection and hyperparameter tuning. SMOTE and weighted loss used together have thereby efficiently balanced the class imbalances and improved the accuracy of the minority class to 88.2% while ensuring robust performance across all categories. Comparative analysis with baseline models like ResNet-50, VGG-16, and XGBoost was presented about the superiority of HDL-ACO in balancing performance with computational efficiency for real-time use in clinical applications.

The modular design and adaptability of the framework to different datasets make it scalable for various diagnostic tasks. Despite the computational complexity and limitations of the dataset, results show that HDL-ACO can be a key transformer for clinical diagnostics. It was capable of improving the diagnostic accuracy and reducing the computational overhead, thus helping health care providers in the early diagnosis of retinal diseases by OCT images. Future work will further fine-tune the ACO algorithm, extend the framework to the other medical imaging modalities, and incorporate interpretability techniques that promote its usability in practice. The HDL-ACO framework is a step toward improving intelligent diagnostic systems, bridging the gap between advanced computational methods and real-world needs for healthcare.

## Data Availability

The datasets used in this study include publicly available image databases. The high-resolution JPEG OCT and chest X-ray images are available in the public Mendeley Data repository at https://doi.org/10.17632/rscbjbr9sj.3. Further data analysed during this study are available from the corresponding author upon reasonable request.
