# Publisher Correction: HDL-ACO hybrid deep learning and ant colony optimization for ocular optical coherence tomography image classification

**DOI:** 10.1038/s41598-025-93294-w

**Published:** 2025-03-12

**Authors:** Shivani Agarwal, Anand Kumar Dohare, Pranshu Saxena, Jagendra Singh, Indrasen Singh, Umesh Kumar Sahu

**Affiliations:** 1 Department of Information Technology, Ajay Kumar Garg Engineering College, Ghaziabad, India; 2 Department of Information Technology, Greater Noida Institute of Technology (Engg. Institute), Greater Noida, India; 3 School of Computer Science Engineering & Technology, Bennett University, Greater Noida, India; 4 School of Electronics Engineering, Vellore Institute of Technology, Vellore, Tamil Nadu 632014 India; 5 Department of Mechatronics, Manipal Institute of Technology, Manipal Academy of Higher Education, Manipal, Karnataka 576104 India

Correction to: *Scientific Reports* 10.1038/s41598-025-89961-7, published online 18 February 2025

In the original version of this Article Pranshu Saxena was incorrectly affiliated with ‘Department of Information Technology, Ajay Kumar Garg Engineering College, Ghaziabad, India’.

The correct affiliation is listed below.

School of Computer Science Engineering & Technology, Bennett University, Greater Noida, India.

The original Article has been corrected.

